# Adult *Loa loa* Filarial Worm in the Anterior Chamber of the Eye: A First Report from Savanna Belt of Northern Nigeria

**DOI:** 10.1371/journal.pntd.0004436

**Published:** 2016-04-07

**Authors:** Sadiq Hassan, Mohammed Isyaku, Abdulsalam Yayo, Farouq Sarkin Fada, Gabriel U. Ihesiulor, Garba Iliyasu

**Affiliations:** 1 Department of Ophthalmology, Faculty of Clinical Sciences, College of Health Sciences, Bayero University Kano, Kano State, Nigeria; 2 Department of Medical Microbiology and Parasitology, Faculty of Clinical Sciences, College of Health Sciences, Bayero University Kano, Kano State, Nigeria; 3 Infectious Disease Unit, Department of Medicine, Faculty of Clinical Sciences, College of Health Sciences, Bayero University Kano, Kano State, Nigeria; Hospital Infantil de Mexico Federico Gomez, UNITED STATES

## Introduction

Filarial worms are tissue-dwelling nematodes that can infest man. The adult filarial parasites, depending on the species, may live in lymphatics, blood vessels, serous membranes, skin, or connective tissues [1]. The adult *Loa loa* worm lives commonly in the connective tissues, eyelids, and the subconjunctiva, where it can be seen moving across the conjunctival sac [2]. Instances of intraocular occurrence of the adult filarial worms are rare, and the few cases which were reported earlier were in the presence of *Wuchereria bancrofti* in either the vitreous or anterior chamber [3,4]. Ocular loiasis caused by adult *L*. *loa* in the subconjunctival space and anterior chamber have been reported in the rainforest areas of Nigeria [5,6,7]. To our knowledge, this is the first report of adult *L*. *loa* worm in the anterior chamber from the savannah belt of northern Nigeria.

## Case Presentation

A 25-year-old woman from Sayaya village in Katsina State, northwestern Nigeria, presented to our eye clinic with a three-month history of redness, pain, photophobia, tearing, and itching, and a two-month history of gradual loss of vision with a sensation of a moving object in the left eye. There was no history of fever, itching, or swelling in any part of the body. She resides in a village which is located close to a hydro-irrigation dam and has never traveled out of the savanna belt of northern Nigeria. She is a full-time housewife, while her husband is a farmer. She had visited a couple of primary health care centres over the last four months and was prescribed topical eye medications with no improvement.

Examination revealed a healthy-looking but anxious patient without subcutaneous nodules or swelling on any part of the body, suggesting no lymphadenopathy. Chest and heart were essentially normal, and there was no hepatosplenomegaly.

**Fig 1:**
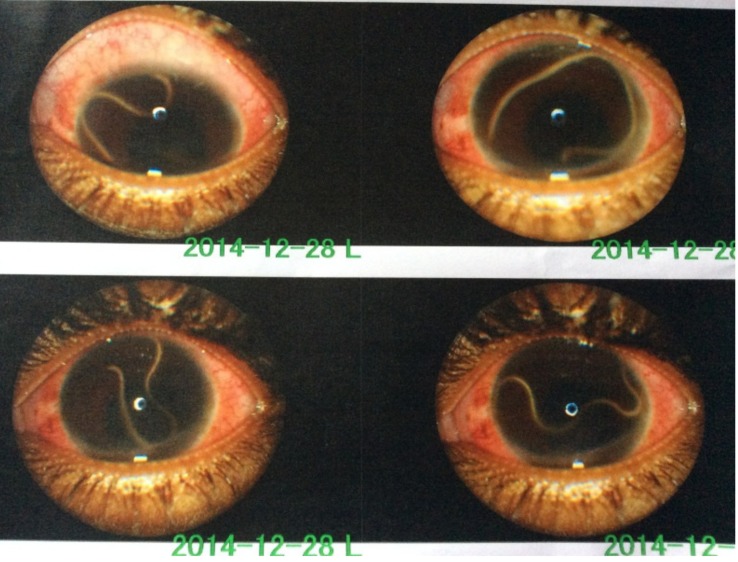
Live adult *Loa loa* worm in the anterior chamber. Examination of the patient’s eye reveals a live adult *L*. *loa* in the anterior chamber.

The left eye examination revealed a visual acuity of light perception, conjunctival hyperemia, a clear cornea, and a live and active thread-like worm in the anterior chamber (Fig 1). The anterior chamber was lined by fibrinous membrane that occluded the pupil. The pupil was irregular, with 360 degree posterior synechia that was associated with a cataract. No abnormality was found in the surrounding ocular adnexa. Right eye examination revealed a visual acuity of 6/5 and normal anterior segment. Intraocular pressure was 12 mmHg and 14 mmHg in the right and left eyes, respectively, using pulse air tonometry. Ocular B-Scan showed vitreous opacities in the left eye.

**Fig 2:**
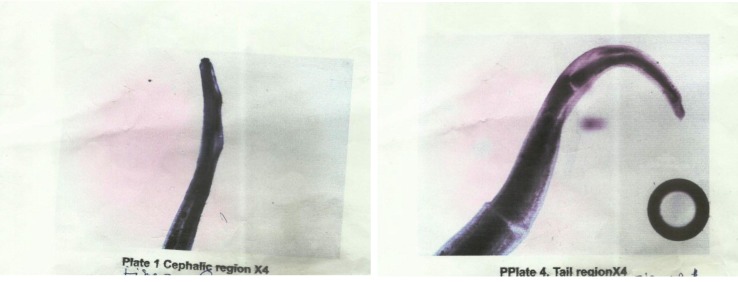
Male adult *L*. *loa*. The worm specimen was fixed in carnoy, stained with Giemsa and haemotoxylin, and examined under a microscope, revealing the presence of a funnel-shaped mouth in the anterior region and the ventral curvature of the tail region.

Full blood count revealed 52% eosinophilia, but multiple midday samples of peripheral blood were negative for microfilaria, and no skin snip examination was done. Stool microscopy did not show any ova or parasites. The patient was admitted for surgical removal of the worm after obtaining consent. While the patient was under local retrobular anesthesia with 2% xylocaine, an operating microscope was used to remove the fully intact worm from the anterior chamber through a superotemporal limbal stab incision. Subconjunctival injections of dexamethasone and ofloxacin were administered four times per day and atropine eye drops 0.5% twice daily. The doses of topical steroid were tapered down over two months. Laboratory analysis of the worm specimen (fixed in carnoy and stained with Giemsa and haemotoxylin), measured 2.3 cm by 0.4 mm. Microscopic examination revealed the presence of a funnel-shaped mouth in the anterior region and the ventral curvature of the tail region (Fig 2). These morphologic taxonomic features are characteristic for adult male *L*. *loa*. The size of the worm was less than the normal range of 3.0–3.4 cm for an adult male *L*. *loa*; this may be explained by the shrinkage that could have occurred prior to microscopic examination.

The patient was treated with oral Albendazole 400 mg daily for 21 days and was followed up closely at the outpatient clinic. The vision in the affected eye deteriorated to no perception of light.

## Case Discussion

Human loiasis is endemic in the rainforest areas of Central and West Africa. Nigeria is one of the countries endemic for *L*. *loa*, where transmission is confined generally to areas in the southeastern and southwestern forest ecological zones. These areas coincide with the geographical distribution of *Chrysops* vector species [11]. The forest ecosystem is a natural habitat for *Chrysops* flies: they rest in the forest canopy and are attracted to man by movement, dark colors, and wood smoke. The infective microfilaria of *L*. *loa* burrows into the human skin when the *Chrysops* vector takes blood meal, but the route of entry of the adult worm into the eye is not known. However, there were speculations that the maturation of the worm from the larval stage might take place inside the eye or that the adult might penetrate the scleral coat of the eye [3,4]. Surprisingly, however, despite the endemicity of loiasis in the forest areas of Nigeria, only a few cases of occurrence of the adult worm in the anterior chamber have been reported [6,7]. Cases of filarial worm *L*. *loa* in the anterior chamber of the eye have, however, been reported in West Africa and other parts of the world [8,9,10]. The presentation of the condition in a patient who has never travelled beyond the savannah ecological belt where her village is located is epidemiologically unexpected. Nevertheless, the village lies in the vicinity of a dam and a large irrigation project, which may provide a local environment optimum for breeding of the *Chrysops* vector. The village could be a neglected local focus of transmission of *L*. *loa* [12].

Detailed microscopic examination of the worm was carried out to differentiate it from *W*. *bancrofti* and *Onchocerca volvulus*, which are endemic in the savannah belt of Nigeria. The severity of the clinical outcome of the condition, resulting in blindness, corroborates the previous reports on cases of intraocular parasitism by adult worm *L*. *loa* in Nigeria [6,7].

**Key Learning Points**

- This report indicates the possibility of occurrence of sporadic cases of loiasis in the communities living in the savanna belt of northern Nigeria.
- We strongly suggest the need to carry out an epidemiological study and *Chrysops* vector surveillance in the savanna belt of northern Nigeria.
- Ophthalmologists in this region should also consider intraocular loiasis when dealing with a case of uveitis or idiopathic intraocular inflammation.
